# Obstetrical and epidemiological factors influence the severity of anal incontinence after obstetric anal sphincter injury

**DOI:** 10.1186/s12884-019-2238-2

**Published:** 2019-03-14

**Authors:** France Joris, Irene Hoesli, Andre Kind, Jean Jacques Ries, Tilemachos Kavvadias

**Affiliations:** Departement of Obstetrics and Gynecology, University Hospital of Basel, Basel, Switzerland

**Keywords:** Obstetric anal sphincter injury, Anal incontinence, Perineal tears, Childbirth

## Abstract

**Background:**

Obstetric anal sphincter injury (OASI) is one of the most severe obstetrical complications. Although risk factors for OASI have been identified, little is known about various parameters that can influence symptoms’ severity. The aim of this study is to explore whether obstetrical and epidemiological factors can have an effect on the severity of symptoms after OASI.

**Methods:**

11.483 deliveries between January 2010 and December 2014 were reviewed, and data from 88 women with OASI are presented.

**Results:**

The only statistically significant differences between symptomatic and asymptomatic women were age (*p* = 0.02), body mass index (*p* = 0.04) and the use of forceps (p = 0.04). Women with more severe symptoms were more likely to have received oxytocin during the second stage of labor (*p* = 0.03) and had shorter delivery to follow-up interval (*p* = 0.008).

**Conclusions:**

Modifiable factors such as use of forceps and oxytocin should be taken into consideration in clinical practice.

## Background

Obstetric anal sphincter injury (OASI) is one of the most severe obstetrical complications and can occur in up to 5.9% of vaginal deliveries [1]. It is the major cause of anal incontinence in young women and also a cause of major morbidity post-partum, both at short and long term, with a positive correlation with bladder, bowel and sexual dysfunction [2, 3]. Anal incontinence, the most severe symptom after OASI, has been reported with a mean prevalence of 39% (15–61%) [4], a fact that contributes to a massive impairment in the quality of life of affected patients [5]. Correct diagnosis and repair of the injury is of utmost important, since the persistence of a sphincter defect can predict anorectal symptoms in the long term [6].

Although there are no accurate methods to predict which patients will experience an OASI at delivery, nulliparity, operative vaginal delivery and infant birthweight > 3500 g, have been consistently identified as independent risk factors for OASI [7, 8]. However, little is known about the different obstetrical and epidemiological factors that could have an influence on established symptoms of women who already have had a sphincter injury. The aim of this study is to detect differences in the severity of anal incontinence according to epidemiological and obstetrical factors.

## Methods

The study was approved by the local ethics committee (Ethikkommission Nordwestschweiz protocol number 2016–01477). Data from all vaginal deliveries > 22 weeks of pregnancy, which took place between January 2010 and December 2014 were retrospectively collected. The study was conducted in a university obstetrical unit in central Europe with a case load of approximately 2500 births per year. Physicians and midwives, both attend to women with vaginal deliveries. According to international recommendations, a standardised care for the protection of the perineum during second stage of labor with a hands-on approach [9]. Episiotomy is not being performed routinely, but in only in cases when a perineal tear is clearly imminent and a difficult instrumental delivery is expected. In the case of OASI, the repair is always performed by an experienced senior physician, who is supervised by a consultant. The end-to-end repair is being preferred and performed as a standard in our institution.

All women who are diagnosed with OASI at birth are enrolled in a programme for pelvic floor rehabilitation and are referred to our special urogynaecology unit not earlier than 6 weeks after delivery. The enrolment is determined just after birth during the 2 days of the hospital stay. There, the initial assessment includes taking medical history regarding pelvic floor symptoms and the completion of the Holschneider-modified Kelly questionnaire for the assessment of anal incontinence [10], a validated questionnaire, which is used to assess the symptoms of anal incontinence through a score which varies from 0 (worse) to 20 (no symptoms). A gynaecological and pelvic examination and as well as an endoanal ultrasound, in which the anal sphincter is scanned for possible defects, and the thickness of the internal and external sphincter is measured, complete the assessment. Women are counselled, and further actions such as physiotherapy or plans for a secondary repair are undertaken if needed.

All women who suffered from OASI after delivery were identified through from the hospital’s patient lists. Patients’ data were extracted by two of the authors (F.J, T.K.), according to the local ethics committee’s regulations. Women who did not accept the enrolment to the pelvic floor rehabilitation programme were excluded from the analysis. Epidemiological data, such as age, parity, nationality and body mass index (BMI), as well as obstetric data, like birth weight, use of oxytocin and/or epidural analgesia/anaesthesia, duration of the second stage of labour and fetal position, were obtained from the medical records.

**Fig. 1:**
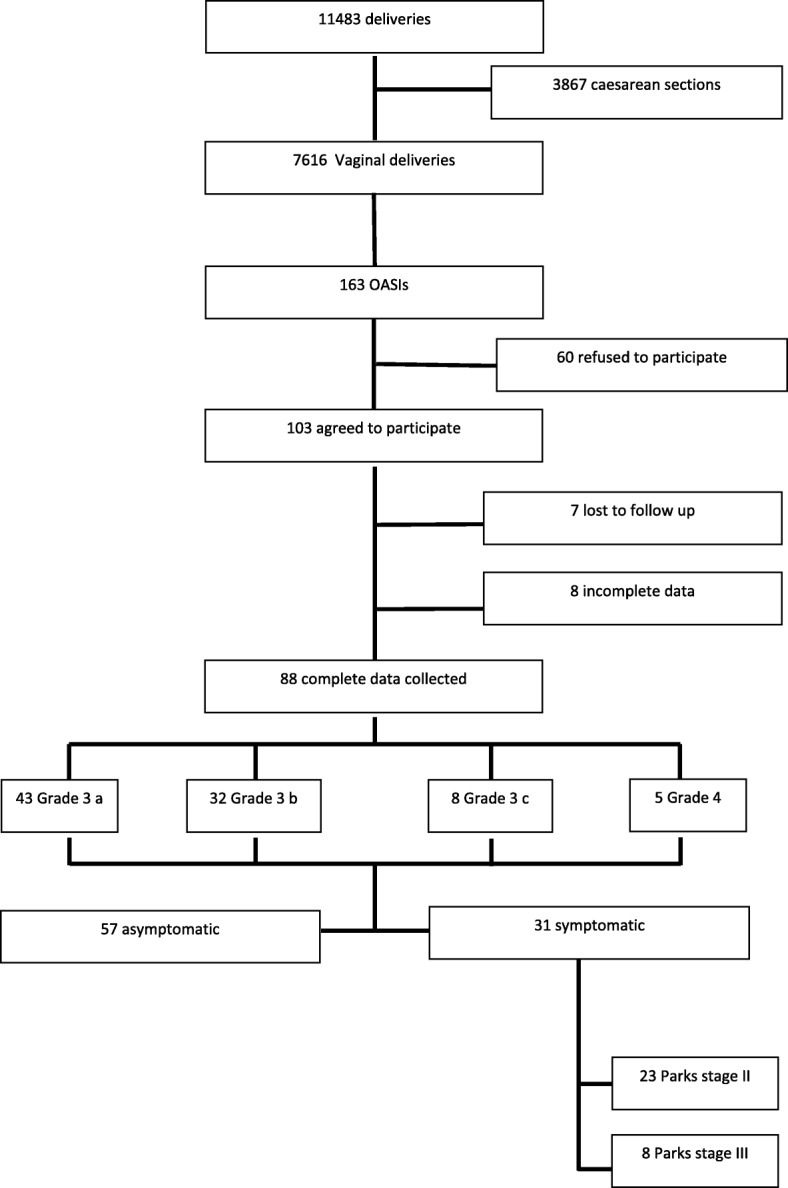
Flow-chart of our patient cohort. (OASI = obstetric anal sphincter injury)

Women were classified into groups according to the standardised Parks classification system for anal incontinence: Parks I: fully continent, Parks II incontinent for flatus, but continent for solid or liquid stool, Parks III: incontinent for liquid stool or flatus but continent for solid stool and Parks IV: incontinent to solid and liquid stool [11]. The initial assessment was performed between asymptomatic and symptomatic women (Parks I-III) and the secondary assessment between the four different Parks groups (see also Fig. 1). For the statistical analysis the R© Statistical Package V. 3.2.5 was used (https://www.r-project.org/). Differences between means were calculated using the Mann-Whitney U-test, whereas for categorical data the Fisher’s exact test was used. The Z-Score was used for the population proportions. The level of statistical significance was set to 0.05.

## Results

Between January 2010 and December 2014, 11,483 deliveries took place at our institution, of which 7616 were vaginal deliveries. Of all vaginal deliveries 163 women (2.14%) were diagnosed with OASI. Out of this group, 103 (63%) agreed to participate and visited our urogynaecology unit after birth. Finally, data from 88 (54%) patients could be collected. From these patients, 31 (35.2%) were symptomatic at the time of the follow up, which was 27.1 (+/− 2.5) weeks for the symptomatic group. Figure 1 describes a detailed flow-chart of patients ‘recruitment and data collection.

Comparing symptomatic with asymptomatic women, we found that symptomatic women were significantly older (32.5 +/− 3.0 vs. 30.4 +/− 4.4, *p* = 0.02) and had a significantly lower body mass index (26.9 +/− 3.5 vs. 28.6 +/− 4.4, *p* = 0.04). The overall rate of vacuum/forceps assisted delivery was not different between the groups (51% vs. 54%, *p* = 0.7), but the use of forceps was significantly higher in the symptomatic group (9.7% vs. 1.8%, p = 0.04). There were no statistically significant differences regarding birth weight (3428 +/− 397 g vs. 3378 +/− 426 g, *p* = 0.5), use of episiotomy (45% vs. 40%, *p* = 0.6), nulliparity at delivery (67% vs. 54%, *p* = 0.2), duration of 2nd stage of labor (104.3 +/− 62.8 vs. 98.9 +/− 74.0 min, *p* = 0.7) and use of oxytocin during 2nd stage of labor (66% vs. 67%, *p* = 0.8).) There were also no statistically significant differences in the postpartum clinical and sonographic assessment of the internal and external anal sphincter as well as no detected sphincter defects. Table 1 shows in detail the comparison between the two groups.

**Table 1:** Comparison of main patients’ characteristics and obstetrical data between symptomatic and asymptomatic women who suffered from obstetric anal sphincter injury during delivery

	Symptomatic, *N* = 31	Asymptomatic, *N* = 57	*p* value
Patients’ characteristics			
Age* (years)	32.5 ± 3.0	30.4 ± 4.4	0.02
Body mass index*(kg/m^2^)	26.9 ± 3.5	28.6 ± 4.4	0.01
Gestational diabetes (%)	6	7	0.6
Obstetrical data			
Weeks of pregnancy*	39.1 ± 1.3	39.4 ± 1.3	0.62
Birth weight (g)*	3428.8 ± 397.8	3378.3 ± 426.6	0.5
Instrumental delivery (%)	51	54	0.5
- Forceps (%)	9.7	1.8	0.04
- Fetal distress ^a^ (%)	43.7	48.3	0.4
Non-occiput anterior presentation (%)	16	17	0.6
2nd stage of labor (min)*	104.3 ± 62.8	98.9 ± 76.0	0.5
Episiotomy (%)	45	49	0.4
Nulliparous at delivery (%)	67	54	0.1
Induced labor (%)	22	24	0.5
Oxytocin during 2nd stage of labor (%)	67	66	0.5
Epidural anaesthesia (%)	58	66	0.2
Estimated blood loss (ml)*	498.3 (166.7)	548.2 ± 402.6	0.2
Male infant (%)	61	52	0.3
Degree of OASI (%)			
3a	38	54	0.3
3b	45	33	0.4
3c	10	7	0.7
4	7	6	0.6
Follow-up assessment			
Follow-up interval in weeks*	27.1 ± 2.5	28.5 ± 48.1	0.6
IAS-width (mm)*	1.5 ± 0.4	1.5 ± 0.5	0.8
EAS-width (mm)*	5.0 ± 1.7	5.5 ± 2.1	0.3
Perineum length (cm) *	2.3 ± 0.6	2.2 ± 0.7	0.5
Pelvic floor strength (Oxford scale)*	2.0 ± 0.6	2.1 ± 0.8	0.5
Kelly-Holschneider Score *	18.2 ± 1.5	19.9 ± 1.1	0.2

All values are in means for continuous numerical data or values for discrete data; in brackets the standard deviation and the percentage respectively. For data marked with (*), the Mann-Whitney U test was used to compare means and calculate the *p* values, all others with the Fisher’s exact test

a: fetal distress as indication for instrumental delivery (forceps or vacuum)

From the 31 symptomatic patients, 23 (74%) were classified as having mild incontinence (Parks stage II, incontinence for flatus) and 8 (26%) as having moderate symptoms (Parks stage III, incontinence for solid stool). No patient presented with complete incontinence (Parks stage IV) (Fig. 1). When women with different severity of anal incontinence were compared, those with more severe symptoms were significantly more likely to have received oxytocin during 2nd stage of labor (100% vs. 56%, *p* = 0.03), had a shorter delivery to follow-up interval (16.2 +/− 2.1 vs. 30.9 +/− 3.4, *p* = 0.008) and scored significantly lower in the Kelly-Holschneider questionnaire (16.7 vs. 18.7, *p* = 0.001) (lower = worse). Symptomatic women also were more likely to have had a non-occiput anterior infant cephalic presentation (37% vs. 9%) and an episiotomy (60% vs. 39%), however these differences as well as other parameters were not significant (Table 2).

**Table 2:** Differences in patients’ characteristics and obstetrical data between women with different severity of anal incontinence (Parks classification) who suffered from obstetric anal sphincter injury during delivery

	Parks stage III, *n* = 8	Parks stage II, *n* = 23	*p* value
Patients’characteristics			
Age*(years)	31.6 ± 4.9	32.8 ± 2.3	0.3
Body mass index* (kg/m^2^)	27.2 ± 2.9	26.8 ± 3.7	0.2
Gestational diabetes (%)	0	9	0.2
Obstetrical data			
Weeks of pregnancy*	38.6 ± 1.6	39.3 ± 1.2	0.1
Birth weight (g)*	3316.8 ± 381.6	3467.1 ± 412.9	0.08
Instrumental delivery (%)	60	48	0.2
Non-occiput anterior presentation (%)	37	9	0.06
2nd stage of labor (min)*	98.9 ± 76.0	104.3 ± 62.8	0.5
Episiotomy (%)	60	39	0.1
Nulliparous at delivery (%)	75	65	0.2
Induced labor (%)	37	21	0.2
Oxytocin during 2nd stage of labor (%)	100	56	0.02
Epidural anaesthesia (%)	78	48	0.3
Estimated blood loss (ml)*	512.5 ± 196.4	493.4 ± 154.8	0.2
Male infant (%)	65	65	0.2
Degree of OASI (%)			
3a	37	39	0.1
3b	25	53	0.2
3c	13	8	0.4
4	25	0	0.08
Follow-up assessment			
Follow-up interval in weeks*	16.2 ± 4.5	30.9 ± 26.5	< 0.001
IAS-width (mm)*	1.6 ± 0.3	1.5 ± 0.5	0.2
EAS-width (mm)*	4.8 ± 1.1	5.0 ± 1.9	0.2
Perineum length (cm) *	2.2 ± 0.7	2.3 ± 0.7	0.2
Pelvic floor strength (Oxford scale)*	1.9 ± 0.6	2.0 ± 0.7	0.3
Kelly-Holschneider Score*	16.7 ± 1.6	18.7 ± 1.1	0.01

All values are in means for continuous numerical data or values for discrete data; in brackets the standard deviation and the percentage, respectively. For data marked with (*) the Mann-Whitney U test was used to compare means and calculate the *p* values, all others with the Fischer’s exact test

Women with higher degree of sphincter defect were more often symptomatic, but the difference was not statistically significant (Table 3).

**Table 3:** Distribution of OASI (obstetric anal sphincter injury) among the symptomatic and asymptomatic women as well as according to the severity of anal incontinence

	Degree of OASI				
Patients	3a	3b	3c	4	Total
Symptomatic (%)	12 (38)	14 (45)	3 (10)	2 (7)	31
Asymptomatic (%)	31 (54)	19 (33)	4 (7)	3 (6)	57
Total OASIS					87
*chi-square 2.23, p = 0.5*					
Parks Stage					
II-mild (%)	9 (39)	12 (53)	2 (8)	0 (0)	23
III-moderate (%)	3 (37)	2 (25)	1 (13)	2 (25)	8
Total symptomatic					31
*chi-square 6.81, p = 0.07*					

## Discussion

The aim of this study was to compare various epidemiological and obstetrical data among women with and without symptoms, but also between groups of women with different symptoms’ severity, after experiencing an OASI. At the time of the writing, and to the best knowledge of the authors, there are no published studies which attempted this approach.

The most interesting finding was that symptomatic women were significantly older. The association between maternal age and the risk for pelvic injury, with or without anal sphincter impairment, has been excessively investigated. Bowling et al., examined the hypothesis that the female pelvis is under structural development until the age of 24 and this could have an influence in the rates of OASI in different age groups [12]. They reviewed the charts of 5937 deliveries but they could not find any association between age and OASI rates. However, other studies have reported some kind of correlation between age and OASI. Ampt et al. report that women > 30 years old have an increased, yet not always statistically significant risk for OASI [13]. Gurol-Urganci et al., in a retrospective study including a huge cohort of 1,035,253 women between 2000 and 2012, found that a higher risk of third- or fourth-degree perineal tears was associated with a maternal age above 25 years [14].

Published data from Rahmanou et al., showed prospectively, that increasing maternal age at first birth is positive correlated not only with diagnosed OASIs, but also with clinically covert pelvic floor damage, which can be revealed post-partum with sonographic imaging [15]. The authors suggested reduced tissue elasticity with advancing age, which might be due to compromised elastic fibre function, as a possible explanation for this positive correlation [16]. This hypothesis could explain our findings as a result of an impaired tissue regeneration process, which is part of the natural tissue ageing process [17, 18]. Available data from other research areas of post-traumatic anal sphincter repair results support this fact [19], but larger prospective studies are needed to examine this possible correlation.

Asymptomatic women in this study also had a higher body mass index at the time of delivery. This fact complements the knowledge that overweight and obesity are associated with an almost linear decrease in the risk of OASI [20]. This has been attributed to the fact, that obese women, having higher serum and myometrial membrane cholesterol levels, which modulates the oxytocin receptor efficacy in uterine smooth muscle [21], are somehow ‘protected’ from an oxytocin overstimulation during the second stage of labor, which subsequently decreases the risk of excessive contractions and pelvic floor injury. Interestingly and on a similar note, in the second part of our analysis, we found that the administration of oxytocin during the second stage of labor is associated with significantly higher symptoms’ severity. This information implies that pelvic floor damage and consequently pelvic floor symptoms are multifactorial: not only are they influenced from known factors such as infant weight at birth or operative delivery but also from oxytocin administration, which, as modifiable factors, could have an impact on obstetrical practice.

Forceps assisted delivery was also more frequent in the symptomatic group (9.7% vs. 1.8%, *p* = 0.04) when compared to the asymptomatic women. The use of forceps has been identified as a risk factor for high degree perineal tears especially when not combined with an episiotomy [22]. However, due to the lack of prospective randomised trials for obvious reasons and due to the fact that use of forceps still remains as a reserve for many obstetricians in the clinical practice, the determination of whether the association of injury to forceps delivery is causal remains unclear. [23]. The fact, though, that there is no difference in the perineal tears when different types of forceps are used could imply such a relationship [24]. The results of the current study also suggest that the overall effect of the forceps delivery on the pelvic floor could be clinically significant, which could suggest a preference, if possible, of vacuum-assisted-deliveries compared to forceps in clinical practise.

Finally, an important finding of the analysis is the fact that, in the symptomatic group, a longer follow up (30.9 vs. 16.2 weeks, *p* = 0.008) was associated with a higher Kelly score and thus with a lower symptom intensity. This finding is consistent with other reports regarding the natural history of symptoms after OASI. Reid et al. report, that out of 31 women with anal incontinence after OASI at 9 weeks, 28 were asymptomatic at 3 years [25]. Similarly, Davé et al. report on a significant reduction of anal incontinence rates and Urogenital Distress Inventory scores after operative vaginal deliveries 3 months after delivery when compared to immediately post-partum [26]. It seems that anorectal symptoms after OASI may show some recovery over time, similar to other pelvic floor symptoms after delivery, such as urinary incontinence [27]. However, it must be noted, that the results of our analysis regarding the different follow up intervals, should be interpreted with caution. Women are being seen in our urogynecological unit after OASI regularly, not earlier than 6 weeks after birth. Unfortunately, it is not possible – due to the work load – to maintain a stable follow-up interval for all patients. Also, as mentioned in the materials and methods section, women agree or refuse to participate before the time of the cross-sectional data collection (i.e. the follow up visit). That said, the most decisive factor for the OASI-follow up interval is the capacity of the outpatient urogynecology unit, a fact however that still cannot eliminate the chronological bias. The ideal design of the study should include a similar follow up intervals in both groups, in order to minimize any possible chronology bias.

This study is not without limitations. Firstly, this is a retrospective analysis, which means that is inevitably prone to bias and the design of the study did not include a statistical power calculation nor a confounding or adjustment. Although the inclusion criteria and the research question were well defined, only 88 out of 163 patients who met these criteria could be included. As a result, there must have been missed data from women who either were symptomatic and did not want to take benefit from the offer or were free from symptoms and decided that a follow up after birth would be unnecessary. In addition, there are possibly important data which are missing from the analysis, such as type of repair, post-partum healing, breast-feeding at the time of the follow-up, smoking habits as well as pelvic floors symptoms before and during pregnancy. Also, the sample size, mostly in the analysis between symptoms’ severity is rather small and a larger sample size could present clearer results. However, the plausibility and the consistency of our findings when compared to previous published studies as stated above, support our statement.

## Conclusions

OASI is one of the most severe complications of vaginal deliveries, since it can have a prolonged negative impact on a woman’s health and quality of life. Correct diagnosis and adequate repair of the injury is crucial, but factors that can influence the presence of symptoms or the symptoms’ severity after OASI should be given adequate attention. The correlation of modifiable factors, such as oxytocin administration during the second stage of labor with OASI should be examined rigorously with prospective studies. Also, considering the fact that maternal age at first birth is rising worldwide, proper counselling of a possible worst outcome after OASI should be considered with increasing maternal age.

## Abbreviation

OASI: Obstetric anal sphincter injury
